# Identifying metabolic biomarkers and pathways in pulpitis: a metabolomic study using ultra-high-performance liquid chromatography/orbitrap mass spectrometry

**DOI:** 10.1590/1678-7757-2024-0428

**Published:** 2025-04-28

**Authors:** Congpeng WEN, Xueqin CHEN, Linfeng LAI

**Affiliations:** 1 Wenzhou Medical University Dingli Clinical College Department of Stomatology Wenzhou Zhejiang China Wenzhou Central Hospital, Wenzhou Medical University, Dingli Clinical College, Department of Stomatology, Wenzhou, Zhejiang, China.

**Keywords:** Pulpitis, Metabolomics, Biomarker, UPLC-Orbitrap/MS, Metabolic pathway

## Abstract

Pulpitis, which is often triggered by caries and trauma, is a significant clinical challenge due to its prevalence. This research aims to uncover potential metabolic biomarkers for pulpitis and map out the implicated metabolic pathways, thereby laying a foundation for enhanced diagnostic and preventive strategies.

**Methodology:**

We analyzed pulp samples from 12 participants (six who had pulpitis and six who had healthy teeth) using serum metabolomics via ultra-high-performance liquid chromatography coupled with Orbitrap mass spectrometry. Important biomarkers were pinpointed via multivariate analysis and orthogonal partial least squares discriminant analysis. Additionally, correlation and biomarker pathway enrichment analyses were conducted to explore the relations between differentially expressed biomarkers and their associated biological pathways. Specific metabolites of interest were further examined via alkaline phosphatase (ALP) staining, Alizarin Red staining, and RT-qPCR analysis.

**Results:**

We identified 22 significant biomarkers (13 increased, nine decreased) related to 18 metabolic pathways in pulpitis cases. Key biomarkers included ascorbic acid, inosine, allopurinol riboside, and L-asparagine, in which ascorbic acid and inosine showed the most substantial downregulation and strongest association with pulpitis. Notably, aminoacyl-tRNA biosynthesis and retrograde endocannabinoid signaling pathways were closely linked with pulpitis. Ascorbic acid enhanced the osteogenic differentiation, calcium deposition, as well as the expression of osteogenic genes of human dental pulp stem cells (DPSCs).Conclusions: The identified biomarkers and metabolic pathways offer insights into the pathogenesis of pulpitis and have potential applications in developing preventive treatments.

## Introduction

Pulpitis, which is a prevalent oral condition, manifests itself as an inflammatory response primarily with pain due to bacterial infections that stimulate the immune system. Often being a consequence of delayed or inadequate management of dental caries, trauma, or other oral health issues, pulpitis shows a notably high incidence rate. Specifically, 88.8% of cases are linked to dental caries, of which 80% require medical intervention. This underscores pulpitis as a significant health concern that needs efficient strategies for prevention and treatment. Despite the availability of advanced treatments that can save the affected tooth, pulpitis leads to irreversible damage. Although preserved, the treated tooth fails to regain its original strength and durability compared to a healthy tooth. This fact accentuates the importance of preventing pulpitis to maintain oral health.^1^

Root canal therapy, which is the standard treatment for pulpitis, alleviates pain and reduces inflammation by removing the pulp and infection. Nevertheless, this method has several drawbacks: it needs pulp extraction, leading to a loss of blood supply, sensory function, and increased tooth fragility; the treatment process inevitably damages dental tissues, diminishing flexural strength and elevating the risk of tooth fracture; it can cause tooth discoloration, impacting on the aesthetics; and despite advancements, the complex nature of the root canal system caps the success rate at about 85%.^2,3^ Moreover, while revascularization for pulp regeneration is clinically employed, its application is limited mainly to young permanent teeth. Consequently, early prevention of pulpitis, maintaining pulp vitality, and prioritizing preventive measures over treatment hold significant clinical importance.

Preventive strategies for pulpitis have largely focused on the prevention and management of dental caries via measures such as oral hygiene, dental fluoridation, sealing pits and fissures, and filling cavities. To date, no preventive strategies targeting pulpitis have been documented. While the prospect of identifying specific genes and proteins to prevent pulpitis seems impractical, small molecules—including peptides and metabolites of low molecular weight—can penetrate cells without eliciting adverse reactions and have been successfully employed in various medical fields. For instance, Ghosh, et al.^4^ (2021) reported a strong association between the generation of intestinal microbial metabolites and their significant effects on the intestinal barrier function and immune response, mediated by interactions with intestinal epithelial cells. Furthermore, peptides derived from BMP-2, such as the OP peptide BMP-2 residues 32-48 and 73-92, have been shown to enhance the MSC levels in RUNX2 and related alkaline phosphatase (ALP) proteins, indicating a role for BMP-2-derived peptides in promoting osteogenic differentiation.^5-7^Consequently, small molecules may represent a promising therapeutic approach for the prevention of pulpitis.

Metabolomics, as a field, significantly advances the identification of disease markers and metabolites with biological activity by both qualitatively and quantitatively analyzing low-molecular-weight metabolites within cells or organisms under various conditions.^8^ This approach is invaluable in diagnosing a broad spectrum of disorders,^9^including neuropsychiatric diseases,^10^ liver fibrosis,^11^ and hepatoma,^12^ and is pivotal in assessing metabolites for disease-causing risks. Furthermore, metabolomics plays a crucial role in predicting cardiovascular diseases, such as hyperlipidemia and acute coronary syndrome,^13-16^ and finds extensive use in dentistry. For instance, Sakanaka, et al.^17^ (2021) uncovered a salivary metabolite profile that is an indicative of cardiac metabolism changes, linking periodontal inflammation to liver function and lipid metabolism disorders. Similarly, Garcia-Contreras, et al.^18^(2015) explored metabolic profile alterations due to TiO2 nanoparticles in gingivitis models, whereas Nijakowski, et al.^19^ (2022) highlighted saliva’s potential for early oral squamous cell carcinoma diagnosis. Moreover, Awaad, et al.^20^ (2017) discovered a novel terpenoid metabolite via Aspergillus terreus extract fractionation, showing significant anti-microbial effects against oral infections. Despite such advances, studies focused on the application of metabolomics to common oral conditions such as pulpitis remain limited, underscoring the need for further research in pulpitis prevention.^21,22^

In this study, we aimed to explore the variances in pulp metabolites between teeth affected by pulpitis and healthy teeth by identifying differentially expressed metabolites (DEMs) with potential utility in preventing pulpitis. To achieve this, we analyzed pulp samples from both pulpitis-affected and healthy teeth employing liquid chromatography-mass spectrometry (LC-MS) techniques to pinpoint specific metabolites. Subsequent statistical analyses helped in distinguishing metabolites that showed altered expression levels in teeth with pulpitis compared to healthy counterparts, laying the groundwork for theoretical strategies in pulpitis prevention.

## Methodology

### Equipment and materials

We used a tabletop high-speed refrigerated centrifuge (TGL-16MS, Lu Xiang Yi Centrifuge Instrument Co., Ltd., Shanghai, China), an ultrasonic cleaning machine (F-060SD, Fuyang Technology Group Co., Ltd., Shenzhen, China), a high-performance liquid chromatography equipment (Nexera UPLC, Shimadzu, Japan), and a high-resolution mass spectrometer (QE, Thermo Fisher Technologies, Cleveland, OH, USA) for analysis.

### Sample collection

Patients who underwent impacted tooth extraction at the Department of Stomatology, Wenzhou Central Hospital from January to May 2023 were enrolled in this study. The study included six patients with normal impacted teeth (healthy dental pulp group) and six with pulpitis (the pulpitis group). After extraction, the impacted teeth were immersed in physiological saline and immediately transported to the laboratory. After cleaning the periodontal tissue attached to the root surfaces of the collected specimens on an ultra-clean table, the root tips were clipped, and the pulp was removed. Thus, 12 sample groups were obtained, which were rapidly stored in liquid nitrogen until further analysis. Patient data, including genetic, family, and medical histories were collected upon admission (Table 1).

**Table 1:** Specimens characteristics from patients.

No.	Gender/Age	Tooth Position	Hight (cm)	Weight (kg)	BMI	B.P. (mmHg)	B.G. (mmol/L)
1	M/44	38	166	70	25.403	139/89	6.09
2	F/38	48	157	55	22.313	127/87	5.44
3	F/57	48	156	68	27.942	132/89	4.77
4	M/48	48	160	73	28.516	129/83	5.05
5	F/37	48	156	69	28.353	135/99	4.66
6	M/35	38	164	84	31.231	132/79	4.28
7	F/38	38	150	50	22.222	113/62	4.34
8	F/47	48	157	70	28.399	118/75	5.01
9	F/53	48	160	70	27.344	130/70	5.21
10	F/27	38	154	74	31.203	120/78	5.39
11	F/36	38	155	53	22.060	120/90	4.82
12	F/48	48	158	63	25.236	133/83	5.51

No.1-6: healthy dental pulp group; No.7-1: the pulpitis group; BMI: Body Mass Index; B.P., Blood Pressure; B.G., Blood Glucose

The inclusion criteria for the pulpitis group were as follows: patients aged between 18 and 60 years; all affected teeth were diagnosed with acute or chronic pulpitis by dental and pulp specialists according to clinical and imaging examinations; caries were the source of the pulp infection; the pulp could be completely removed; the patients had not taken any medication within the past three months. The exclusion criteria for pulp tissue samples from healthy teeth without caries were as follows: patients with serious brain, heart, kidney, liver, or endocrine system diseases; an association between the affected tooth and severe periodontitis. Clinical personnel monitored the baseline during sample collection to ensure that the samples within each group remain within a stable range. Additionally, dietary consumption was standardized prior to sample collection to ensure that the results of the metabolomics analysis were not influenced by dietary variations.

The study design was approved by the Ethical Committee of Wenzhou Central Hospital, China. The registration number is 202406100850000154127. Further, all participants provided informed consent prior to sample collection. All experiments were performed in accordance with the declaration of Helsinki.

### Sample preparation and analysis

After inactivation, all the dental pulp samples were subjected to 30 min sterilization at 56°C. Thereafter, equivalent amounts of samples (100 μL) were placed in centrifuge tubes of 1.5 mL, followed by adding 0.3 mg/mL of L-2-chlorolphenylalanine (10 μL) and 0.01 mg/mL of LysoPC17:0 in methanol as the endogenous standard under 10 s vortexing. Then, a pre-chilled methanol-acetonitrile mixture (300 μL, v:v=2:1) was added during 1-min vortexing, followed by 10-min ultrasonication at 0°C and 30-min incubation at -20°C. After 15-min centrifugation at 13,000 rpm and 4°C, supernatant samples were collected in new centrifuge tubes and dried using a freeze-concentration centrifugal dryer. Lyophilization was then performed. The powder samples were then re-dissolved in a mixed methanol-water solution (100 μL, v:v=1:4), followed by 30-s vortexing and 2-min standing at 4°C. The resultant mixed sample was then subjected to 5-min centrifugation at 13,000 rpm and 4°C to collect supernatants, which were then filtered using 0.22-μm microfilters into LC vials before LC-MS analysis. The supernatant samples (200 μL) were added to the glass sampling vials and enabled to dry at 25°C under vacuum conditions, followed by the addition of methoxylamine hydrochloride (15 mg/mL) in pyridine (80 μL). The resulting mixed samples were subjected to 2-min vigorous vortexing and 90-min incubation at 37°C, after which BSTFA containing 1% TMCS (80 μL) and n-hexane (20 μL) was added.

The mixture was then subjected to 2-min vortexing and 60-min derivatization at 70°C followed by incubation for 30 min at 25°C prior to LC-MS analysis. To prepare the quality control (QC) sample, equal volumes of diverse sample extracts were mixed, with the same QC volume as the sample volume. A QC sample was prepared by mixing equal volumes of the extraction solutions from all samples. During the analysis process, a QC sample was interspersed after every six to eight samples. The stability of the instrument during the analysis was then assessed based on the consistency of the QC samples (PCA plot of the QC). The RSD screening of the QC samples involved statistical analysis of the relative quantification values of each ion peak in the QC samples, with the removal of ion peaks that showed a high RSD.

### Untargeted metabolomics analysis based on LC-MS

The Acquity UHPLC system (Waters Corporation, Milford, USA) coupled with an AB SCIEX Triple TOF 5600 System (AB SCIEX, Framingham, MA, USA), functioning in the electrospray ionization of positive- and negative-ion modes was used to obtain plasma metabolic profiles. We then employed the Waters BEH C18 column (1.71 m, 2.1×100 mm) (Waters Corporation, Milford, MA, USA), with the mobile phase containing water (supplemented with 0.1% formic acid) and acetonitrile for chromatography. The analysis conditions were as follows: injection volume, 1 μL; column temperature, 45°C; and flow rate, 0.4 mL/min. Furthermore, data were obtained under the full-scan mode at 70-1,000 m/z and in the IDA mode, with the collision energy at 30 eV and m/z range at 25-1,000. To evaluate data repeatability, QC samples were loaded at 10-run intervals. Moreover, Progenesis QI software (Waters Corporation) was utilized to analyze the raw LC-MS data using the following parameters: fragment tolerance, 10 ppm; precursor tolerance, 5 ppm; noise elimination level, 10.00; and retention time (RT) tolerance, 0.02 min. During the data analysis, we eliminated isotopic peaks, with the minimal intensity set at 15% of the base peak intensity. Additionally, LIPID MAPS (https://lipidmaps.org/), HMDB (https://hmdb.ca/), along with self-built databases, were adopted for metabolite identification based on tandem mass spectrometry (MS/MS) spectra and RT-m/z pairs. Finally, we exported the output data matrix, which included 3-D datasets (m/z, peak RT, and intensity data) for subsequent analyses.

### Metabolic pathway enrichment

The DEMs were subjected to metabolic pathway enrichment analysis to elucidate the mechanisms underlying the alterations of metabolic pathways within normal and pulpitis samples. The enriched metabolic pathways were determined based on the Kyoto Encyclopedia of Genes and Genomes (KEGG) database.

### Osteoblast differentiation and mineralization

Human dental pulp stem cells (DPSCs) were initially seeded into 48 Well Cell Culture Plates at a density of 5 × 10⁴ cells/well and let it adhere for 24 h. The cells were then cultured in a commercial osteogenic differentiation medium supplemented with 100 μg/mL guanosine or N-α-acetyl-L-asparagine. The medium was refreshed twice a week. On days seven and 21, alkaline phosphatase (ALP)-positive cells were detected by using the BCIP/NBT kit (Beyotime, Shanghai, China), and extracellular matrix calcium deposition was visualized by using 1% Alizarin Red S solution (Solarbio, Beijing, China).

### Quantitative PCR assay

**Figure 1:**
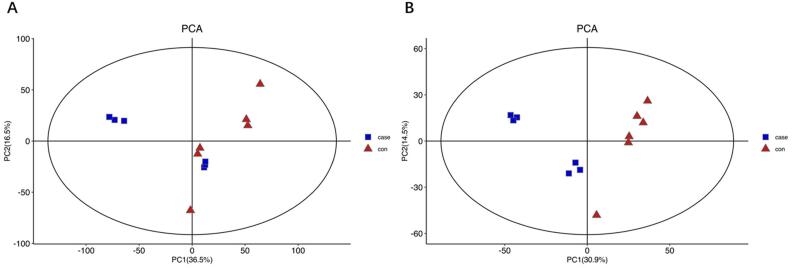
Score plot of principal component analysis (PCA). Closed triangle, control (six samples); closed square, pulpitis group (six samples) (A) Score plot of the PCA model in the positive mode; (B) Score plot of the PCA model in the negative mode.

Osteogenic gene expression is reportedly necessary for treating pulpitis^23^. Thus, to evaluate the effect of inosine and ascorbic acid on osteogenesis-related gene expression, human DPSCs were seeded in 6 Well Cell Culture Plates at a density of 5×10^5^ cells/well, then co-cultured with 100 μg/mL guanosine or N-α-acetyl-L-asparagine for 72 h. Thus, the function of target metabolites was preliminarily verified. The total RNA was extracted from samples by using the Axygen RNA Miniprep Kit (Axygen, Union City, CA, USA) according to the manufacturer’s instructions. To obtain cDNA, total RNA was reverse transcribed with the Prime Script RT reagent Kit (Takara Biotechnology, Otsu, Shiga, Japan). Next, a real-time quantitative PCR (qPCR) assay was performed on an ABI 7500 Sequencing Detection System (Applied Biosystems, Foster City, CA). A 10-μL reaction system of SYBR^®^ Premix Ex Taq™ II (Takara Biotechnology, Otsu, Shiga, Japan) was established by mixing 5 μL of TB Green, 3 μL of ddH_2_O, 1 μL of cDNA, 0.4 μL of each primer and 0.2 μL of ROX Dye 2. The reaction system was amplified for 40 cycles (95 ℃ for 5 s and 60 ℃ for 30 s for each cycle). Melting curves were verified to ensure the specificity of amplification. The gene expression that is relative to control was calculated using the comparative 2^CT^method. The *Gapdh enzyme* was considered as the housekeeping gene. Figure 1 shows the primer sequences.

### Statistical analysis

#### Using Thermos Xcalibur 2.2 software

Peaks were aligned and extracted by the Thermo Xcalibur 2.2 software (Thermo Fisher Scientific, San Jose, CA, USA). Thus, we obtained a data table containing RT-m/z values and adjusted *p*-values corresponding to the data of the peak area.

#### Multivariate analysis

To observe the sample distribution and analyze the process stability, unsupervised principal component analysis (PCA) was performed for multivariate analysis using the Simca P software version 13.0 (Umetrics, Umea, Sweden). Thereafter, the intergroup heterogeneities of the metabolic profiles and DEMs were analyzed via supervised orthogonal partial least squares discriminant analysis (OPLS-DA).

Furthermore, we screened the variables with the variable importance in the projection (VIP) values above 1.0 and determined two-tailed *p*-values via Student’s *t*-tests using the SPSS software version 18.0.0 (IBM, Armonk, NY, USA). Statistical significance was set at *p*<0.05.

#### Univariate analysis

We performed univariate analysis to describe the centralized and discrete trends of the samples. We applied univariate statistics to infer the overall situation based on the obtained sample data. This included a comparison of metabolites based on statistical hypothesis testing and interval estimation via Student’s *t*-test as well as fold change (FC) analysis. Further, both FC and *p*-values, which facilitated DEM selection, were visualized using volcano maps.

#### Differential metabolite screening

We used VIP to select differentially expressed metabolites as it facilitates the measurement of impact intensity and has the explanatory ability of THE metabolite expression patterns to classify, discriminate, mine biologically significant DEMs, and verify their significance using the *t*-test. Significant DEMs and the top 50 differential metabolites with VIP values v>1.0 showed hierarchical clustering.

#### Correlation analysis

A correlation analysis based on Pearson’s correlation coefficients was performed to evaluate the relations between the DEMs and biological pathways. In addition, we performed a correlation analysis to determine the degree of linear correlation between two DEMs.

## Results

### Metabolite screening

The Compound Discoverer software (Thermo Fisher Scientific) identified 22 differentially expressed biomarkers, including 13 upregulated and nine downregulated, associated with 18 major metabolic pathways. Age, sex, body mass index, and other indicators (Table 2) did not differ significantly between the two groups (*p*>0.05).

**Table 2:** Differentially expressed metabolites.

m/z	Retention time (min)	Ion mode	Metabolites	Mass Error (ppm)	VIP	p-Value	log2 (FC)	Compound ID
1.750.242	1.11	neg	Ascorbic acid	-3.39	2.18	3.40E-03	-3.34	HMDB0000044
2.670.739	2.23	neg	Inosine	1.60	2.58	5.42E-03	-1.50	HMDB0000195
2.690.887	2.26	pos	Allopurinol riboside	2.50	2.95	8.10E-03	-1.27	HMDB0000481
1.330.612	0.72	pos	L-Asparagine	3.37	1.11	1.26E-02	-1.23	HMDB0000168
8.345.303	13.37	neg	PE(18:3(6Z,9Z,12Z)/22:4(7Z,10Z,13Z,16Z))	1.54	3.12	5.33E-04	-1.07	HMDB0009141
3.590.486	1.13	neg	5′-Phosphoribosyl-N-formylglycinamide	-3.34	1.38	7.72E-03	-0.66	HMDB0001308
2.840.996	2.25	pos	Guanosine	2.22	1.18	3.36E-02	-0.56	HMDB0000133
2.890.683	1.22	neg	Uridine	0.66	4.08	1.94E-02	-0.48	HMDB0000296
1.910.193	1.13	neg	Diketogulonic acid	-3.26	4.23	6.89E-04	-0.33	HMDB0005971
2.000.478	4.18	pos	Tryptophan	3.83	5.88	3.56E-04	0.20	HMDB0003447
1.480.608	0.75	pos	L-Glutamic acid	2.17	2.41	2.51E-02	0.23	HMDB0000148
5.022.942	10.67	pos	Fexofenadine	-1.93	1.94	3.07E-02	0.33	HMDB0005030
1.950.507	0.77	neg	D-Ribose	-2.48	1.14	1.26E-03	0.51	HMDB0000283
5.443.414	10.82	pos	LysoPC(20:4(5Z,8Z,11Z,14Z))	2.99	1.34	2.88E-02	0.55	HMDB0010395
2.031.508	0.86	pos	Asymmetric dimethylarginine	2.79	1.61	1.55E-03	0.69	HMDB0001539
4.803.100	11.11	pos	Dodecanedioylcarnitine	3.17	3.00	4.20E-02	0.98	HMDB0013327
3.101.140	0.82	pos	N-Acetylneuraminic acid	2.35	1.66	1.99E-02	1.02	HMDB0000230
3.601.508	0.82	pos	Inulobiose	2.28	8.87	1.08E-05	1.47	HMDB0029898
1.660.867	2.86	pos	L-Phenylalanine	2.98	6.44	1.15E-04	2.03	HMDB0000159
3.002.904	10.57	pos	3-Dehydrosphinganine	2.34	1.21	3.53E-02	5.74	HMDB0001480
3.283.218	10.96	pos	11Z-Eicosenoic acid	2.44	1.51	4.03E-02	6.54	HMDB0002231
2.800.832	7.41	neg	4-Hydroxyphenylacetylglutamic acid	1.76	1.40	4.94E-02	12.90	HMDB000606

### Biomarker selection and detection

**Figure 2:**
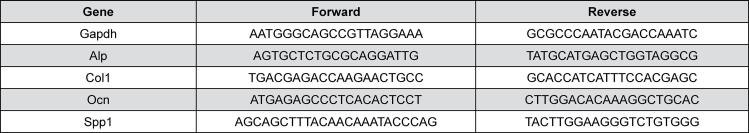
Sequences used in RT-qPCR.

Based on the PCA analysis (Figure 2), an isolation trend was observed within and between the control and pulpitis groups, with the blank controls and pulpitis groups showing better separation trends.

**Figure 3:**
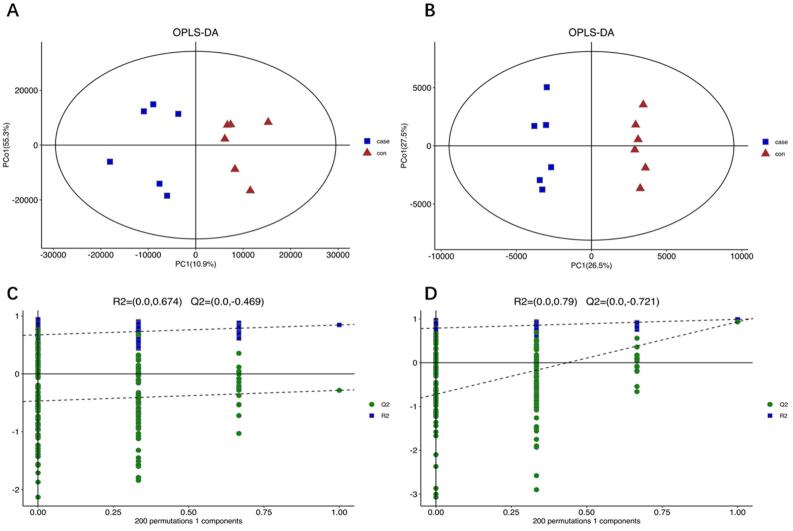
Score plot of the orthogonal partial least squares discriminant analysis (OPLS-DA) model. Closed triangle, control (six samples); closed square, pulpitis group (six samples). (A) Score plot of the OPLS-DA model in the positive mode; (B) Score plot of the OPLS-DA model in the negative mode; (C) Permutation test of the OPLS-DA model in the positive mode; (D) Permutation test of the OPLS-DA model in the negative mode.

An OPLS-DA model was established for biomarker screening. The results corresponding to the pulpitis group were markedly different from those of the control group, suggesting the stability and reliability of the model. Collectively, 22 differentially expressed metabolites satisfying VIP>1 and *p*<0.05 were detected. Among them, ascorbic acid, inosine, allopurinol riboside, asymmetric dimethylarginine, dodecanedioylcarnitine, and N-acetylneuraminic acid showed highly significant changes (Figure 3). That is, asymmetric dimethylarginine, dodecanedioylcarnitine, and N-acetylneuraminic acid were significantly elevated in the pulpitis samples, while ascorbic acid, inosine, and allopurinol riboside were significantly reduced.

**Figure 4:**
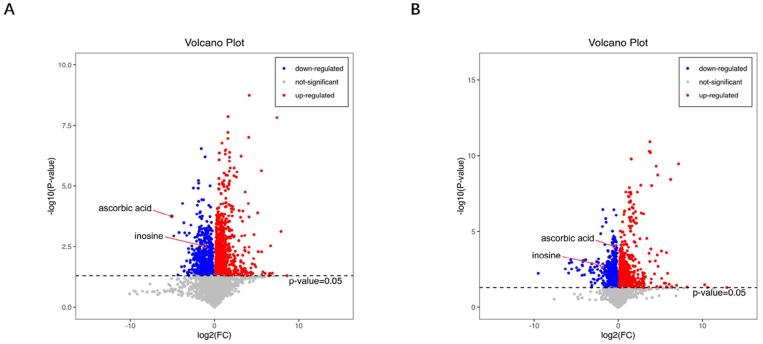
Volcano plot. (A) Core plot of univariate statistical analysis results in the positive mode; (B) Score plot of univariate statistical analysis results in the negative mode. Red and blue origins represent significantly upregulated and downregulated metabolites in the experimental group, respectively; gray points represent non-significant metabolites.

A volcano plot was then generated to visualize the fold change and *p*-values and select DEMs (Figure 4). Samples from the same group appeared in the same cluster, and metabolites within one cluster had similar expression profiles. These observations showed the blank control and blood stasis model groups could be clustered into two categories, indicating that the screened metabolites were reasonable.

**Figure 5:**
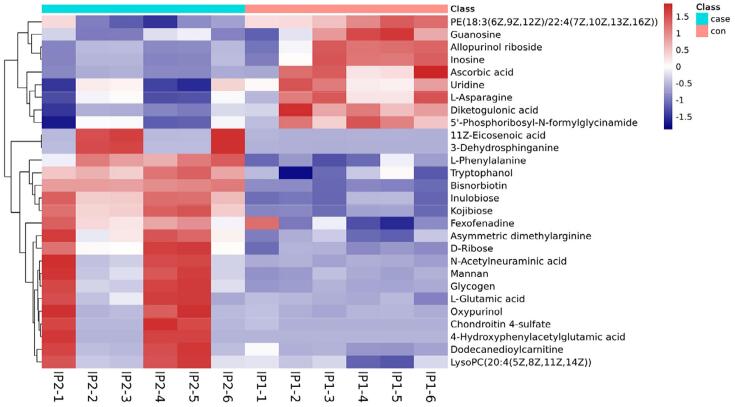
Heat map of differentially expressed metabolites: IP1-1–IP1-6, control groups; IP2-1–IP2-6, pulpitis group. Blue and red color intensity indicates the abundance of metabolites from low to high.

Hierarchical clustering was performed for the significant DEMs. Thus, 50 DEMs with the highest variable importance in the projection values were used to obtain a heat map. After verifying the reasonability and accuracy of the selected DEMs, samples from an identical group was observed to appear in the same cluster, and metabolites within one cluster had similar expression profiles (Figure 5). Moreover, the blank control group and pulpitis group clustered into two categories, further indicating that the screened metabolites were reasonable.

**Figure 6:**
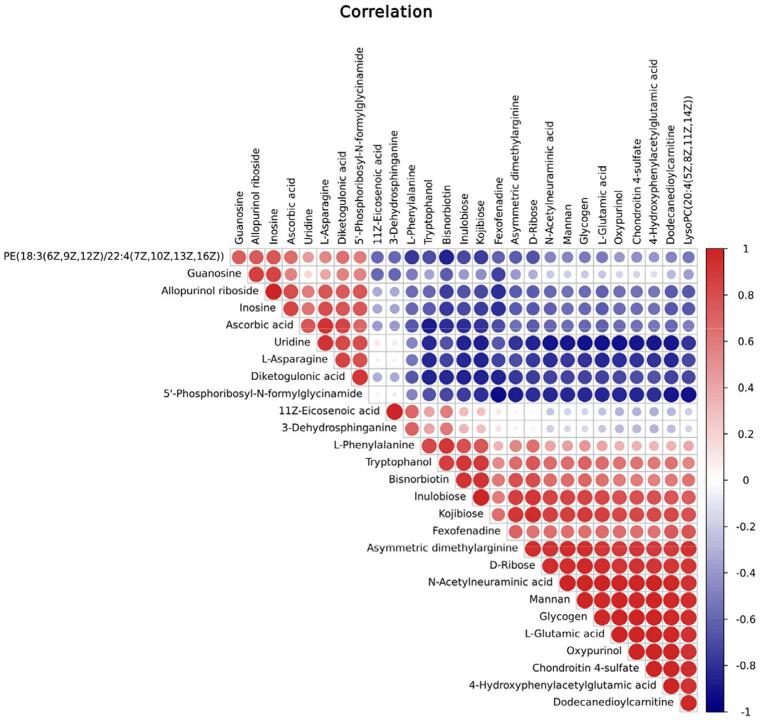
Correlation analysis involving differentially expressed metabolites. Red, positive; blue, negative.

The Pearson’s correlation analysis was performed to determine the degree of linear correlation between two metabolites. The top 50 most significantly altered metabolites with the highest VIP values were selected for visual analysis. Ascorbic acid and inosine were positively correlated with allopurinol riboside, asymmetric dimethylarginine, and dodecanedioylcarnitine, while N-acetylneuraminic acid and ascorbic acid were negatively correlated with tryptophan (Figure 6). Furthermore, inosine showed a negative relation with fexofenadine.

### Biomarker pathway analysis

**Figure 7:**
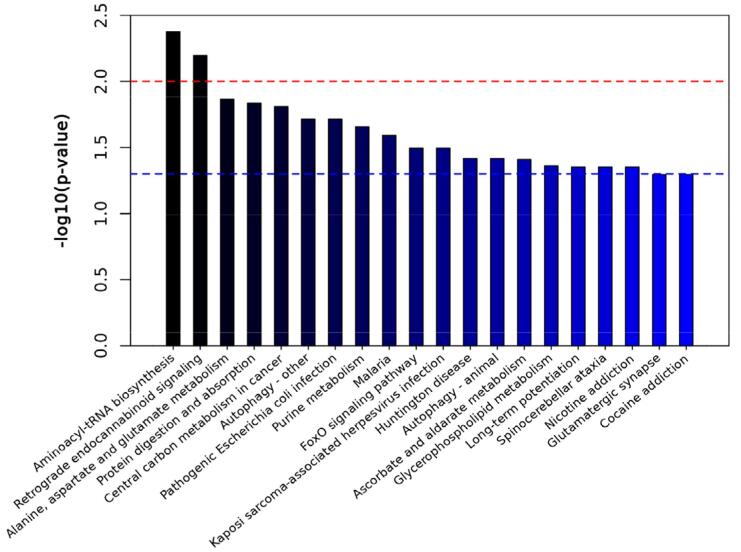
Enrichment map of the top 20 metabolic pathways. Red line, p=0.01; blue line, p<0.05. When the top of the column surpasses the blue line, the signal pathway represented by the p is significant.

The KEGG database was used for the analysis of biomarker pathway enrichment (Figure 7), revealing that the biomarkers were predominantly associated with aminoacyl-tRNA biosynthesis, retrograde endocannabinoid signaling, glutamate, alanine, aspartate metabolism, central carbon metabolism within malignancies, and protein digestion and absorption. Moreover, among these pathways, aminoacyl-tRNA biosynthesis and retrograde endocannabinoid signaling were most significantly correlated with pulpitis.

### Functional verification of metabolites

**Figure 8:**
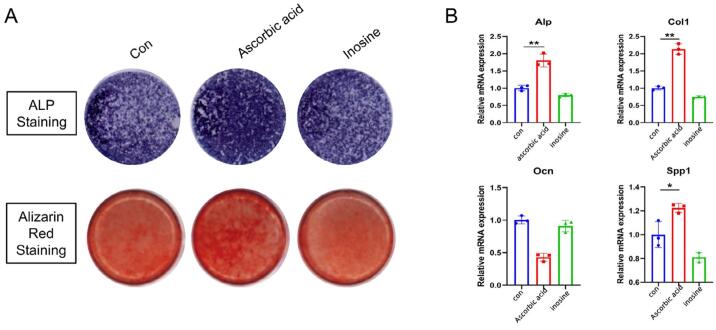
Functional verification of metabolites. A. Representative Alkaline Phosphatase Staining (seven days of osteogenic medium induction) and Alizarin Red Staining (21 days of osteogenic medium induction) of the human dental pulp stem cells. B. Expression of osteogenesis-related genes in human DPSCs evaluated by RT-qPCR. The data are presented as the mean ± SD (*p<0.05, **p<0.01).

We also investigated the effect of ascorbic acid and inosine on osteogenesis. The results of alkaline phosphatase staining demonstrated that ascorbic acid significantly promoted osteogenic differentiation on day seven of induction compared to the control group of DPSCs, while inosine showed no promotive effect. Similarly, Alizarin Red staining revealed that ascorbic acid significantly enhanced calcium deposition on day 21 of osteogenic induction, whereas inosine showed no such effect (Figure 8A). Meanwhile, the transcriptional level of osteogenesis-related factors, including *ALP, COL1,* and *SPP1*, were significantly upregulated following incubation with ascorbic acid, however, were unchanged after inosine incubation (Figure 8B). Such findings indicate that ascorbic acid promoted the expression of osteogenesis-related genes in human dental pulp steam cells, which may promote the restorative process of injured pulp tissue.

## Discussion

Pulpitis results from the invasion of dental pulp tissue by plaque biofilm, leading to inflammation of the pulp tissue. This condition prompts a defensive response from both the dental pulp and surrounding damaged tissues.^23^ Consequently, the metabolic profile of pulpitis-affected tissue markedly diverges from a healthy dental pulp. Although root canal therapy can manage the irreversible damage caused by pulpitis to a significant extent, the development of effective preventive measures is paramount.^24^ In this context, the emerging field of metabolomics offers promising avenues for the innovation of preventive strategies for pulpitis.

Metabolomics, which is defined as the comprehensive study of dynamic metabolic changes within biological systems in response to biological stimuli, pathophysiological states, or genetic variations, serves as an essential branch of systems biology.^25^ It is uniquely positioned to elucidate functional changes in living organisms, thanks to its ability to analyze a wide array of biological samples, including fluids (such as urine, serum, plasma, saliva, bile, and tears), tissues (such as brain tumor and adipose tissues), and cells.^26^ Key analytical techniques in metabolomics encompass chromatography-mass spectrometry methods, including nuclear magnetic resonance, capillary electrophoresis-mass spectrometry (CE-MS), and liquid chromatography-mass spectrometry (LC-MS). Notably, ultra-performance liquid chromatography (UPLC) coupled with atmospheric pressure ionization mass spectrometry (API-MS) stands out for its high sensitivity, dynamic range, and versatility, making it the preferred choice for metabolomic research. Such methodologies enable detailed analysis and evaluation of target metabolites, facilitating the elucidation of their metabolic pathways and functions. Consequently, metabolomics offers foundational insights for the diagnosis and therapeutic intervention of clinical disorders.^27-30^Currently, research on sequencing biomarkers related to periodontitis and caries primarily focuses on inflammatory factors, amino acid products, bacterial products, relevant inorganic ions, and short-chain fatty acids, whereas there is a lack of sequencing studies specifically targeting metabolic products.

In this research, we employed metabolomics to discover metabolites intimately linked with pulpitis, aiming to uncover the disease’s underlying mechanisms and guide the development of prophylactic measures. We utilized untargeted metabolomics via UPLC-Orbitrap/MS to explore potential biomarkers in pulp tissues of individuals afflicted by pulpitis. This analysis revealed 22 biomarkers—thirteen upregulated and nine downregulated—implicated in 18 significant metabolic pathways. Notably, such pathways involve crucial metabolites such as ascorbic acid, inosine, allopurinol riboside, and L-formaldehyde. Our findings indicate a strong association between pulpitis and specific pathways, notably aminoacyl-tRNA biosynthesis and retrograde endocannabinoid signaling.

Ascorbic acid, commonly known as vitamin C, plays a crucial role in the physiological functions of the human body, including its antitumor and antioxidative activities. Despite the human body’s inability to synthesize vitamin C, it can be acquired via dietary sources. Furthermore, its inclusion in antitumor drug formulations acts as a tumor growth inhibitor via various mechanisms such as scavenging reactive oxygen species (ROS) and inducing selective ROS generation, which increases its toxicity towards tumor cells.^31^ As an antioxidant, ascorbic acid combats free radical oxidation, targets cancer cells, and is used in treating cardiovascular diseases (CVDs). Its involvement extends to several physiological processes like collagen synthesis and the repair of tissues, fractured bones, teeth, and cartilage.^32^ This also reduces monocyte adhesion to the endothelium, enhances vasodilation and endothelium-dependent nitric oxide production, and decreases vascular smooth muscle cell apoptosis, thereby preventing the formation of unstable plaques in atherosclerosis.^33^In the context of oral health, ascorbic acid plays a role in modulating the inflammatory responses that are associated with periodontitis and is essential for dentin formation under both pathological and physiological conditions.^34^ Nevertheless, specific reports on ascorbic acid’s effectiveness in pulpitis treatment are scarce. We observed significantly reduced levels of ascorbic acid in the pulpitis group compared to the control, indicating that a deficiency in ascorbic acid might accelerate the necrosis and degeneration of pulp tissue, thereby hastening the progression of pulpitis. This hypothesis is further supported by ALP staining, Alizarin Red staining, and RT-qPCR function verification experiments demonstrating that ascorbic acid enhances osteogenic differentiation, calcium deposition, as well as the expression of human osteogenic genes, thereby facilitating the repair of damaged pulp tissue.

In this research, inosine was pinpointed as a significant metabolite that showed markedly lower levels in the pulpitis group compared to the normal group. Inosine was identified in 1965 as a component of the first sequenced transfer RNA (tRNA), tRNA Ala^35^ and is recognized for its role as the inaugural base modification in nucleic acids. This signifies the formation of purine nucleoside from hypoxanthine, playing a crucial role in the pathways of purine biosynthesis and degradation. Beyond its biochemical pathways, inosine is integral to neuronal signal transmission and is found in various RNAs, impacting their transfection efficiency and accuracy.^36^ As a pivotal secondary metabolite derived from purine degradation, inosine influences the structure and function of exogenous and non-coding RNAs, acting as a molecular messenger in cellular processes.^37^ Inosine may have a critical role in the pathophysiology of pulpitis, and its downregulation can lead to excessive release of inflammatory mediators, exacerbating the inflammatory response.^38^ Additionally, decreased inosine levels may increase cell apoptosis, resulting in pulp tissue damage and further inflammation.^39^ This reduction can impair the pulp’s repair capacity, delaying the healing process. Moreover, inosine downregulation may promote inflammation progression by affecting signaling pathways such as NF-κB. In summary, inosine downregulation may be closely linked to pulpitis progression, influencing inflammation, cell survival, and tissue repair.^40^ The diminished levels of inosine observed in the pulpitis group suggest its potential involvement in exacerbating pulp nerve necrosis and advancing the disease. However, inosine’s influence on osteogenic gene expression minimally appeared, indicating the need for further investigation into inosine and other metabolites showing significant downregulation in pulpitis.

However, as a preliminary study metabolomic testing on pulpitis tissue, this study has several limitations. First, the sample size is small, only composed of 12 participants. Further studies with larger cohorts and individual analyses are needed. Moreover, this study is subject to a series of confounding variables, such as age, gender, BMI, and exercise habits, which need to be considered and controlled in future researches. Furthermore, the functional assays that focus solely on osteogenesis-related genes are limited in this study. In future researches, we will further investigate the functional experiments of metabolites in related fields such as osteoclastogenesis, inflammation, and tissue repair.

## Conclusions

Our findings demonstrate that metabolites that show significant expression changes in pulpitis patients hold potential both as diagnostic markers and therapeutic targets. Notably, ascorbic acid and inosine emerged as critical differential metabolites that are potentially linked to pulpitis pathogenesis. Additionally, our analysis identified aminoacyl-tRNA biosynthesis and retrograde endocannabinoid that signal pathways as intimately connected with pulpitis. Such insights lay the groundwork for future investigations into the interplay between metabolites and pulpitis, aiming to elucidate the disease’s pathogenesis further and foster the creation of efficacious preventive treatments.
